# A New Titanosaurian Braincase from the Cretaceous “Lo Hueco” Locality in Spain Sheds Light on Neuroanatomical Evolution within Titanosauria

**DOI:** 10.1371/journal.pone.0138233

**Published:** 2015-10-07

**Authors:** Fabien Knoll, Lawrence M. Witmer, Ryan C. Ridgely, Francisco Ortega, Jose Luis Sanz

**Affiliations:** 1 School of Earth, Atmospheric & Environmental Sciences, University of Manchester, Manchester, United Kingdom; 2 School of Earth Sciences, University of Bristol, Bristol, United Kingdom; 3 Departamento de Paleobiología, Museo Nacional de Ciencias Naturales-CSIC, Madrid, Spain; 4 Department of Biomedical Sciences, Heritage College of Osteopathic Medicine, Ohio University, Athens, Ohio, United States of America; 5 Departamento de Física Matemática y de Fluidos, Facultad de Ciencias, Universidad Nacional de Educación a Distancia, Madrid, Spain; 6 Departamento de Biología, Facultad de Ciencias, Universidad Autónoma de Madrid, Madrid, Spain; Institute of Botany, CHINA

## Abstract

Despite continuous improvements, our knowledge of the neurocranial anatomy of sauropod dinosaurs as a whole is still poor, which is especially true for titanosaurians even though their postcranial remains are common in many Upper Cretaceous sites worldwide. Here we describe a braincase from the uppermost Cretaceous locality of ‘‘Lo Hueco” in Spain that is one of the most complete titanosaurian braincases found so far in Europe. Although the titanosaurian *Ampelosaurus* sp. is known from the same locality, this specimen is clearly a distinct taxon and presents a number of occipital characters found in *Antarctosaurus* and *Jainosaurus*, which are approximately coeval taxa from southern Gondwana. The specimen was subjected to X-ray computed tomographic (CT) scanning, allowing the generation of 3D renderings of the endocranial cavity enclosing the brain, cranial nerves, and blood vessels, as well as the labyrinth of the inner ear. These findings add considerable knowledge to the field of sauropod paleoneuroanatomy in general and titanosaurian endocast diversity in particular. Compared with that of many sauropodomorphs, the endocast appears only slightly flexed in lateral view and bears similarities (e.g., reduction of the rostral dural expansion) with Gondwanan titanosaurians such as *Jainosaurus*, *Bonatitan*, and *Antarctosaurus*. The vestibular system of the inner ear is somewhat contracted (i.e., the radius of the semicircular canals is small), but less so than expected in derived titanosaurians. However, as far as the new specimen and *Jainosaurus* can be contrasted, and with the necessary caution due to the small sample of comparative data currently available, the two taxa appear more similar to one another in endocast morphology than to other titanosaurians. Recent phylogenetic analyses of titanosaurians have not included virtually any of the taxa under consideration here, and thus the phylogenetic position of the new Spanish titanosaurian—even its generic, let alone specific, identification—is not possible at the moment. Nevertheless, both the braincase osteology and the endocast morphology suggest that the specimen represents a derived titanosaurian that presumably branched further from the base of Lithostrotia, potentially even near Saltasauridae, comparable in evolutionary terms with *Jainosaurus*.

## Introduction

During the construction of a high-speed rail track connecting Madrid with Valencia in 2007, an exceptional fossil site was discovered in the Villalba de la Sierra Formation at a locality named “Lo Hueco,” near the village of Fuentes, Castile-La Mancha, Spain. Over the course of several months, a large-scale excavation yielded thousands of specimens of plants, invertebrates, and vertebrates of late Campanian-early Maastrichtian age [1]. Together with eusuchian crocodiles, titanosaurian sauropods represent the largest part of the fossil record at “Lo Hueco.” Among this rich sauropod collection, only a few cranial elements were collected: two braincases and a small number of isolated teeth. These skull elements are extremely important remains as the cranial anatomy of titanosaurians is currently poorly known globally, except for a few remarkable exceptions (see [2–7]). The same holds true of the neuroanatomy of titanosaurians even though much progress has been achieved lately [8–10]. The aim of the present paper is to present a detailed osteological description together with a paleoneurological investigation of the most completely preserved sauropod braincase from “Lo Hueco”, which is also one of the more complete from the European Upper Cretaceous as a whole. The other titanosaurian braincase from “Lo Hueco” was described previously and referred to *Ampelosaurus* sp. [9]. Although both braincases obviously pertain to titanosaurians, they are morphologically distinct and clearly belong to different taxa.

### Repository abbreviations

CCMGE: Chernyshev’s Central Museum of Geological Exploration, Saint Petersburg, Russia; CM: Carnegie Museum of Natural History, Pittsburgh, United States of America; FAM: Mairie de Fox-Amphoux, Fox-Amphoux, France; FGGUB: Facultatea de Geologie şi Geofizică a Universității din Bucureşti, Bucharest, Romania; GSI: Geological Survey of India, Kolkata, India; ISI: Indian Statistical Institute, Kolkata, India; MACN: Museo Argentino Ciencias Naturales “Bernardino Rivadavia”, Buenos Aires, Argentina; MB.R., Collection of fossil Reptilia, Museum für Naturkunde, Berlin, Germany; MCCM: Museo de las Ciencias de Castilla-La Mancha, Cuenca, Spain; MCF: Museo “Carmen Funes”, Plaza Huincul, Argentina; MCNA: Museo de Ciencias Naturales de Álava, Vitoria, Spain; MDE: Musée des Dinosaures, Espéraza, France; MGPIFD: Museo de Geología y Paleontología del Instituto de Formación Docente Continua, General Roca, Argentina; MNHN: Muséum National d'Histoire Naturelle, Paris, France; MPCA: Museo Provincial “Carlos Ameghino”, Cipolletti, Argentina; PVL: Instituto Miguel Lillo, Tucumán, Argentina; MUCPv: Museo de la Universidad Nacional del Comahue, Neuquén, Argentina.

## Materials and Methods

The August-December 2007 excavations at “Lo Hueco,” in which FK, FO, and JLS took part, were carried out according to the authorization 04-0392-P11 issued by the Dirección General de Patrimonio y Museos of the Junta de Comunidades de Castilla-La Mancha (Toledo, Spain). All necessary permits were obtained, which complied with all regulations.

Sauropods show a wide disparity in skull morphology and titanosaurian braincases are easily distinguished from those of non-titanosaurian taxa (see e.g., [11, 12]). The osteology of the specimen, MCCM-HUE-1667, will be contrasted with that of all the European Late Cretaceous (Campanian-Maastrichtian) titanosaurian specimens reported so far that are sufficiently complete to be informative for comparative purposes, based on the literature and, where necessary, unpublished photographic material and personal observations (Table 1). The paleoneurology of MCCM-HUE-1667 will be compared with all the other titanosaurians for which the endocast (either physical or digital) of the cranial cavity and/or osseous labyrinth has been described (Table 2).

**Table 1:** Titanosaurian braincases used for comparison in this study.

**Specimen**	**Taxon**	**Age**	**Country**
MCNA 7439 ([30]: figs 2–4)	*Lirainosaurus astibiae* Sanz et al., 1999 [55]	Campanian	Spain
MDE C3-76 ([18]: fig. 4.2)	*Ampelosaurus atacis* Le Loeuff, 1995 [56]	Campanian	France
MCCM-HUE-8741 ([9]: figs 1, 2, S1, S2, S3)	*Ampelosaurus* sp.	Campanian or Maastrichtian	Spain
Mechin collection 225 ([23]: unnumb. pl.)	indeterminate	Campanian or Maastrichtian	France
MNHN unnumb. ([19]: fig. 2, pls 5–6)	indeterminate	Campanian	France
FAM 03.064 ([12]: figs 2–5)	indeterminate	Campanian	France
FGGUB 1007 ([20]: fig. 15; [67]: fig. 2.10)	indeterminate	Maastrichtian	Romania

**Table 2:** Titanosaurian endocasts used for comparison in this study (the first column indicates the specimens from which they are derived).

**Specimen**	**Taxon**	**Age**	**Country**
MCCM-HUE-8741 ([9]: figs 1, 2, S1, S2, S3)	*Ampelosaurus* sp.	Campanian or Maastrichtian	Spain
GSI K27/497 ([28]: fig. 7; [38]: fig. 6)	*Jainosaurus septentrionalis* (Huene et Matley, 1933) [38]	Maastrichtian	India
MACN-RN 821 ([8]: figs 1–3, 9A, E; [44]: fig. 13.4, 13.6–13.8, 13.11)	*Bonatitan reigi* Martinelli et Forasiepi, 2004 [68]	Campanian or Maastrichtian	Argentina
MACN 6904 ([8]: figs 4–5, 9B, F)	*Antarctosaurus wichmannianus* Huene, 1929 [21]	Campanian	Argentina
PVL 4017–161 ([8]: fig. 8B)	*Saltasaurus loricatus* Bonaparte et Powell, 1980 [69]	Maastrichtian	Argentina
MGPIFD-GR 118 ([8]: fig. 6, 9D, H; [70]: fig. 4)	indeterminate	Campanian or Maastrichtian	Argentina
MCF-PVPH 765 ([8]: figs 8, 9C, G; [11]: fig. 3)	indeterminate	Coniacian	Argentina

To produce a three-dimensional reconstruction of the endocast of the cranial cavity and endosseous labyrinth of the inner ear, the specimen was subjected to computed tomographic (CT) scanning on an Yxlon CT Compact (Yxlon International, Hamburg, Germany) with a voltage of 180 kV and a current of 2.8 mA. The in-plane pixel size was 0.18 mm, with an inter-slice spacing of 0.20 mm. Data were output from the scanner in DICOM format and then imported into Avizo 7.1 (VSG, Burlington, MA, USA) for digital extraction of the anatomical features of interest. The scan data were difficult to work with due to there being little contrast between the bone and matrix and apparently some scanner-induced artifact that produced banding; nevertheless, the scan data were adequate to segment the major features of the cranial endocast and endosseous labyrinth. The resulting 3D models were then imported into the 3D modeling software Maya 2012 (Autodesk, San Rafael, CA, USA) for removal of segmentation artifacts, final rendering, and generation of the illustrations. The 3D PDF in the Supporting Information (S1 Fig) was generated by exporting the 3D models from Maya into Deep Exploration 5.5 (Right Hemisphere, San Ramon, CA, USA) and then Adobe Acrobat 9 Pro Extended (Adobe Systems Inc., San Jose, CA, USA). The 3D PDF can be viewed with any computer on which even the free Adobe Reader program is installed, allowing the user to work interactively with the product of the digital segmentation to rotate and resize the structures, turn structures on and off, make them transparent, etc. The CT scan data in DICOM file format are freely and publicly accessible at: http://dx.doi.org/10.5061/dryad.dg027

### Systematic Paleontology

Dinosauria Owen, 1842 [13]
Saurischia Seeley, 1888 [14]
Sauropoda Marsh, 1878 [15]
Titanosauria Bonaparte et Coria, 1993 [16]
Lithostrotia Wilson et Upchurch, 2003 [17]
Lithostrotia indet.

## Description

### Osteology

**Fig 1:**
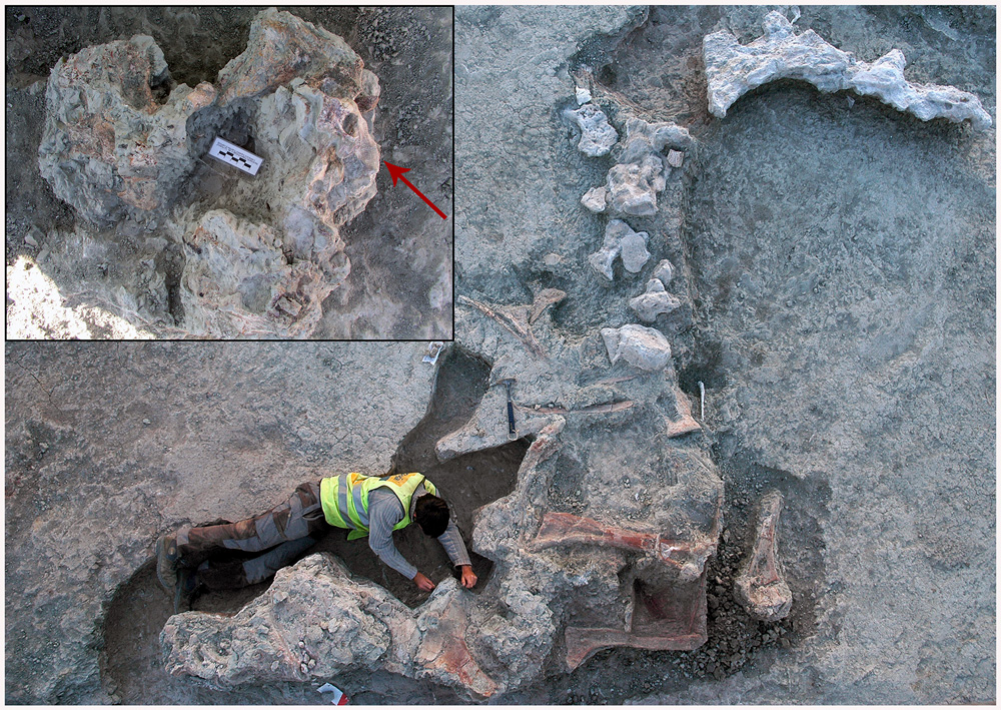
Photograph of the indeterminate titanosaurian sauropod skeleton from the Cretaceous of Fuentes, Spain, with which the braincase MCCM-HUE-1667 was associated. The neurocranium had already been removed when the photo was taken, but the inset (arrow) shows how it appeared in the field, after partial coating with gauze.

**Fig 2:**
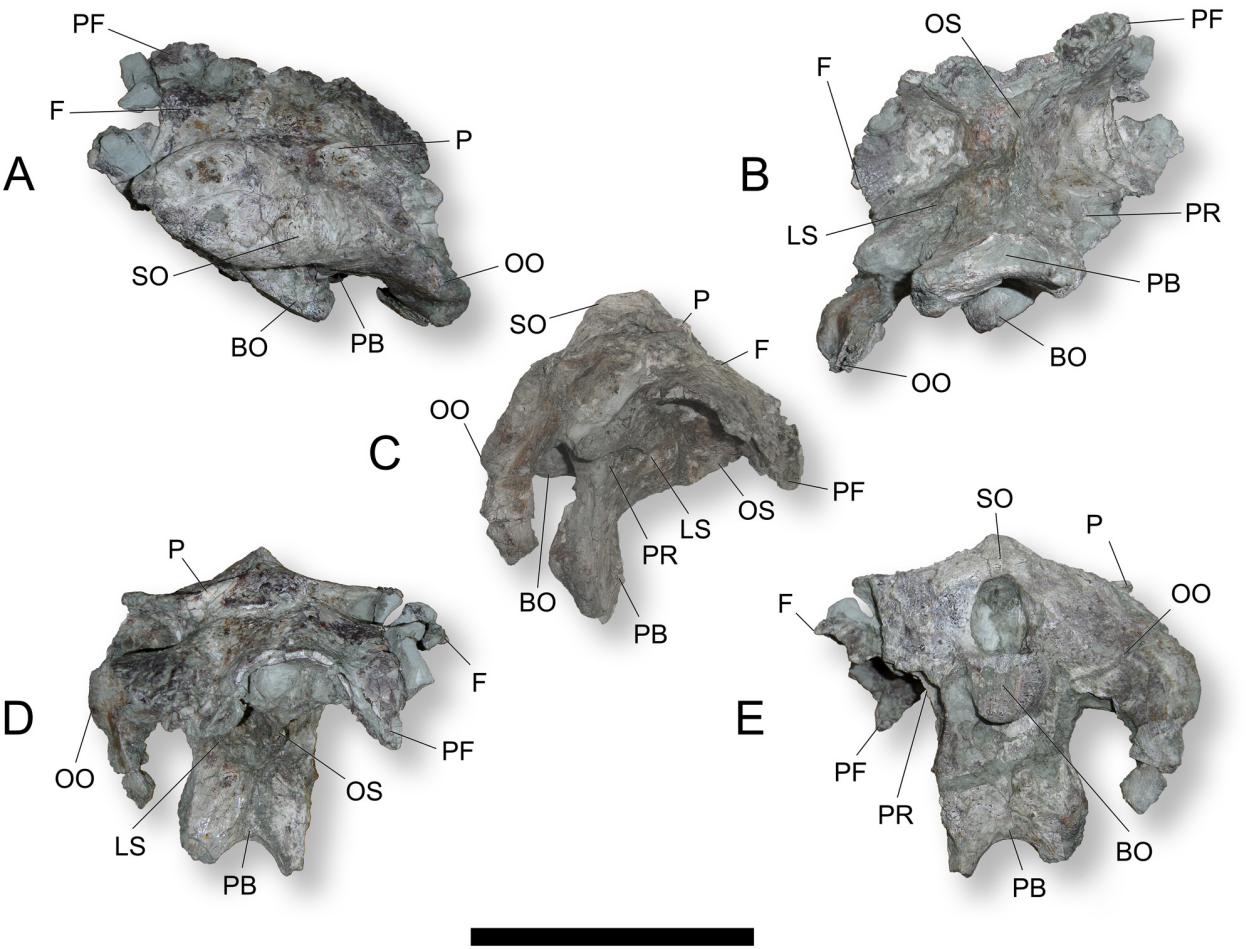
Photographs of the indeterminate titanosaurian sauropod braincase MCCM-HUE-1667 from the Cretaceous of Fuentes, Spain. In dorsal (A), ventral (B), right lateral (C), rostral (D), and caudal (E) views. Rostral is to the top in (A, B), dorsal is to the top in (C-E). Abbreviations: BO, basioccipital; F, frontal; LS, laterosphenoid; OO, otoccipital; OS, orbitosphenoid; P, parietal; PB, parabasisphenoid; PF, prefrontal; PR, prootic; SO, supraoccipital. Scale bar equals 10 cm.

**Fig 3:**
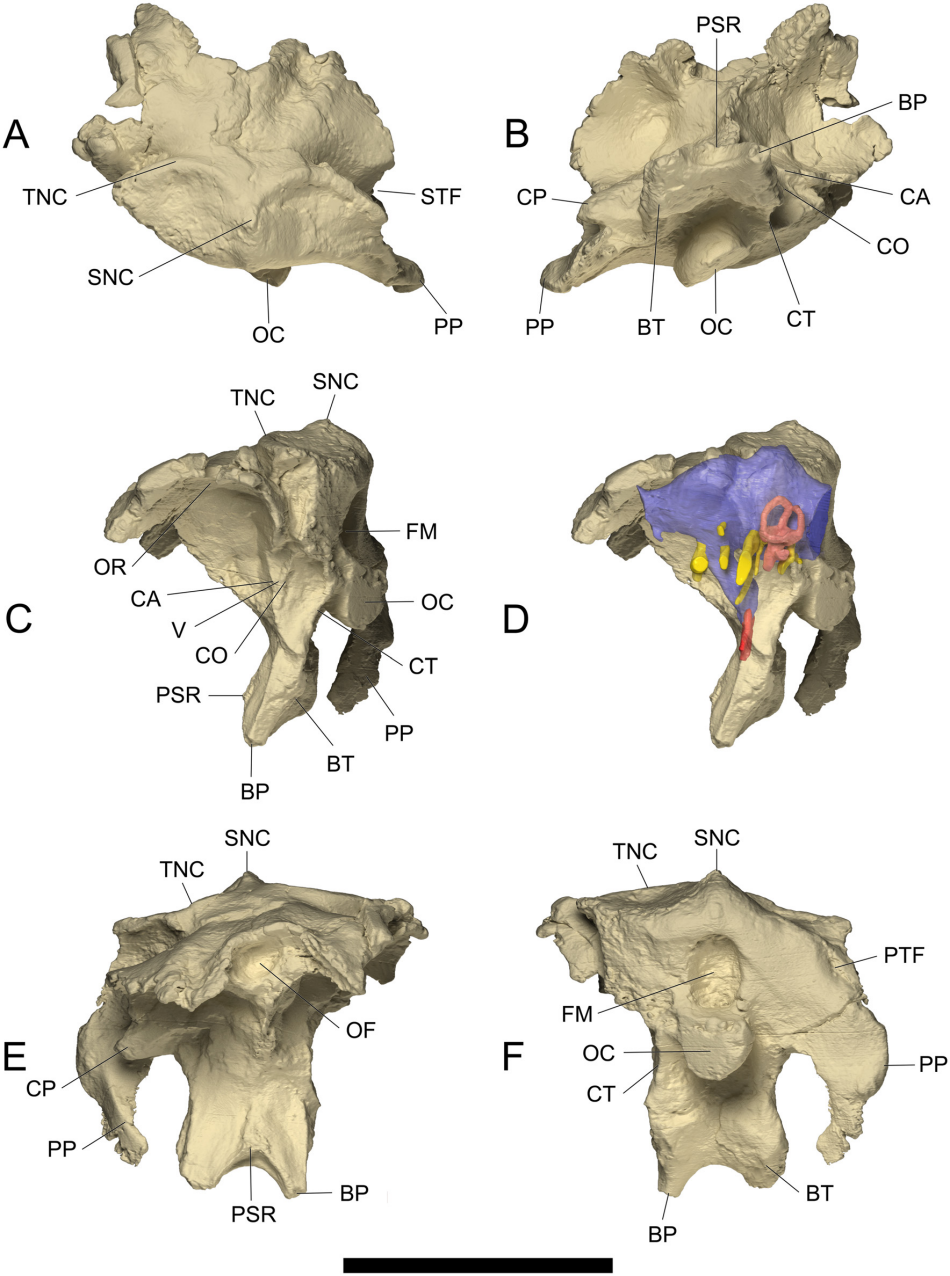
Volume-rendered CT-based images of the indeterminate titanosaurian sauropod braincase MCCM-HUE-1667 from the Cretaceous of Fuentes, Spain. Opaque and unfilled in dorsal (A), ventral (B), left lateral (C), rostral (E), and caudal (F) views and semitransparent with the endocast and associated structures in left lateral view (D). Rostral is to the top in (A, B), dorsal is to the top in (C-F). Abbreviations: BP, basipterygoid process; BT, basal tuber; CP, capitate process; CA, crista antotica; CO, crista otosphenoidea; CT, crista tuberalis; FM, foramen magnum; OC, occipital condyle; OF, olfactory fenestra; OR, orbital recess; PP, paroccipital process; PSR, parasphenoidal rostrum; PTF, posttemporal fenestra; SNC, sagittal nuchal crest; STF, supratemporal fenestra; TNC, transverse nuchal crest; V, trigeminal foramen. Scale bar equals 10 cm.

MCCM-HUE-1667 was discovered in the lowest part of the fossiliferous succession of “Lo Hueco” (G1; see [1] and references therein), in close association with cervical vertebrae of a partial sauropod skeleton (Fig 1). It has been slightly sheared during early diagenesis, fractured by the shrink-swell cycles of clay minerals, and is missing the left caudolateral portion, due to breakage in the field by the bucket of a mechanical digger. As in many other braincases of sauropods in general and titanosaurians in particular, the sutures are generally indistinct. In this specimen, every bone was fully ossified and most elements are fused together. Numerous fractures hinder observation of the braincase surface, but the specimen is otherwise well preserved. Overall, the braincase is short and deep. It is 101.9 mm long rostrocaudally, 127.4 mm tall dorsoventrally, and the better-preserved right side is 67.9 mm wide transversally (Figs 2 and 3, S1 Fig).

#### Prefrontal-Frontal

The frontal is a rostrocaudally short bone (Figs 2A, 2C, 3A and 3C, S1 Fig). We interpret a ventrally curved process (more complete on the left side) that projects from the rostrolateral corner as an articulated prefrontal (Figs 1A–1D, 3A, 3B and 3E, S1 Fig). If this process had been preserved on the right side too, the combined prefrontals-frontals would have a U-shaped rostral margin. The dorsal surface of the frontal is not flat, but corrugated with a channel that runs from the caudomedial area toward the rostrolateral corner. It is probable that the rostromedial fraction of the supratemporal fenestra was formed by the caudal portion of the frontal (although the lack of clear sutures prevents any certainty in this regard). Ventrally, the frontal is markedly concave as it forms the orbital roof (Figs 2B, 2C, 3B and 3C, S1 Fig). The prefrontal projection is buttressed so that it has a triangular outline in cross-section. The transverse breadth of the combined prefrontal-frontal is 57.0 mm at most and extends 41.8 mm rostrocaudally.

**Fig 4:**
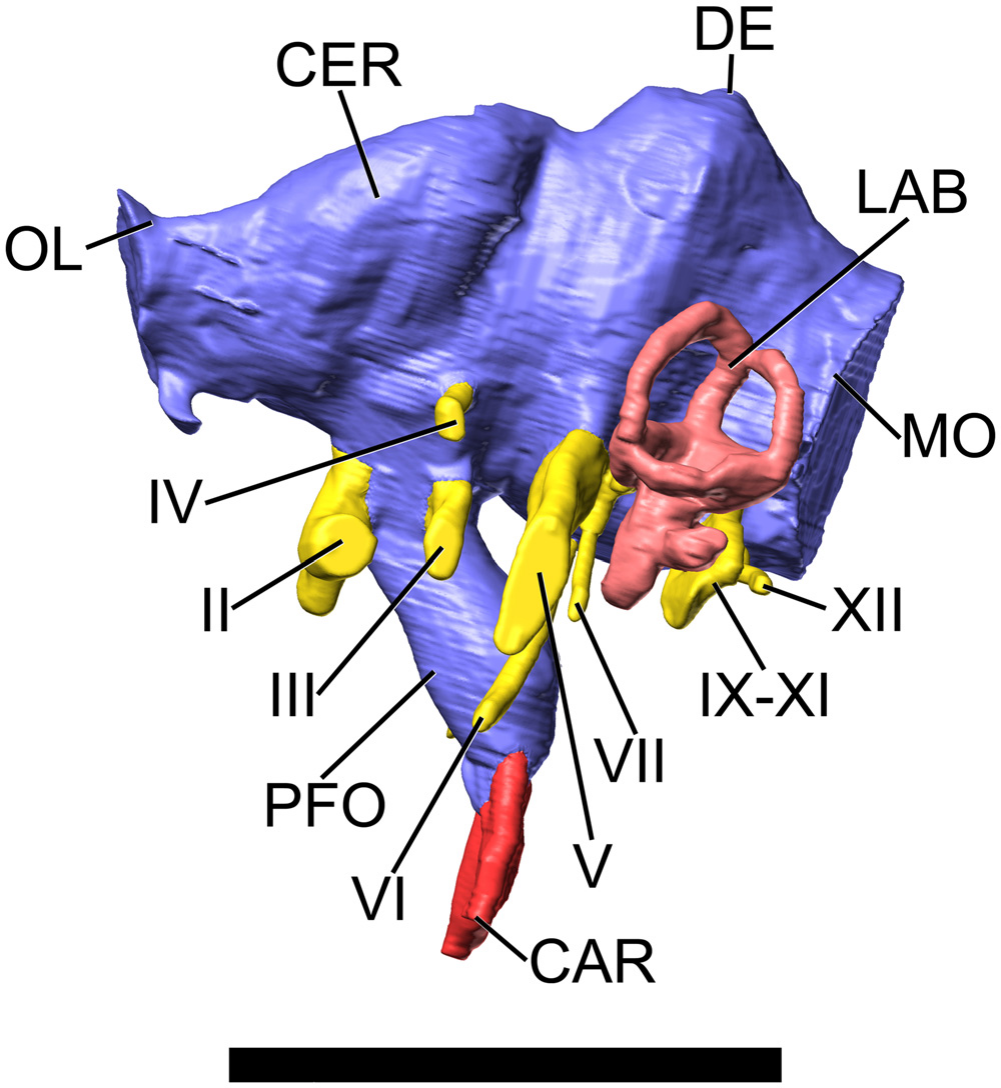
Surface-rendered CT-based images of the cranial endocast and endosseous labyrinth of the indeterminate titanosaurian sauropod braincase MCCM-HUE-1667 from the Cretaceous of Fuentes, Spain. In left lateral view. Dorsal is to the top. Abbreviations: CAR, internal carotid artery; CER, cerebrum; DE, dural expansion; II, optic nerve; III, oculomotor nerve; IV, trochlear nerve; LAB, labyrinth; MO, medulla oblongata; OL, olfactory lobe; PFO, pituitary body; V, trigeminal nerve; VI, abducens nerve; VII, facial nerve; IX-XI, glossopharyngeal and vagoaccessory nerves; XII, hypoglossal nerve. Scale bar equals 5 cm.

**Fig 5:**
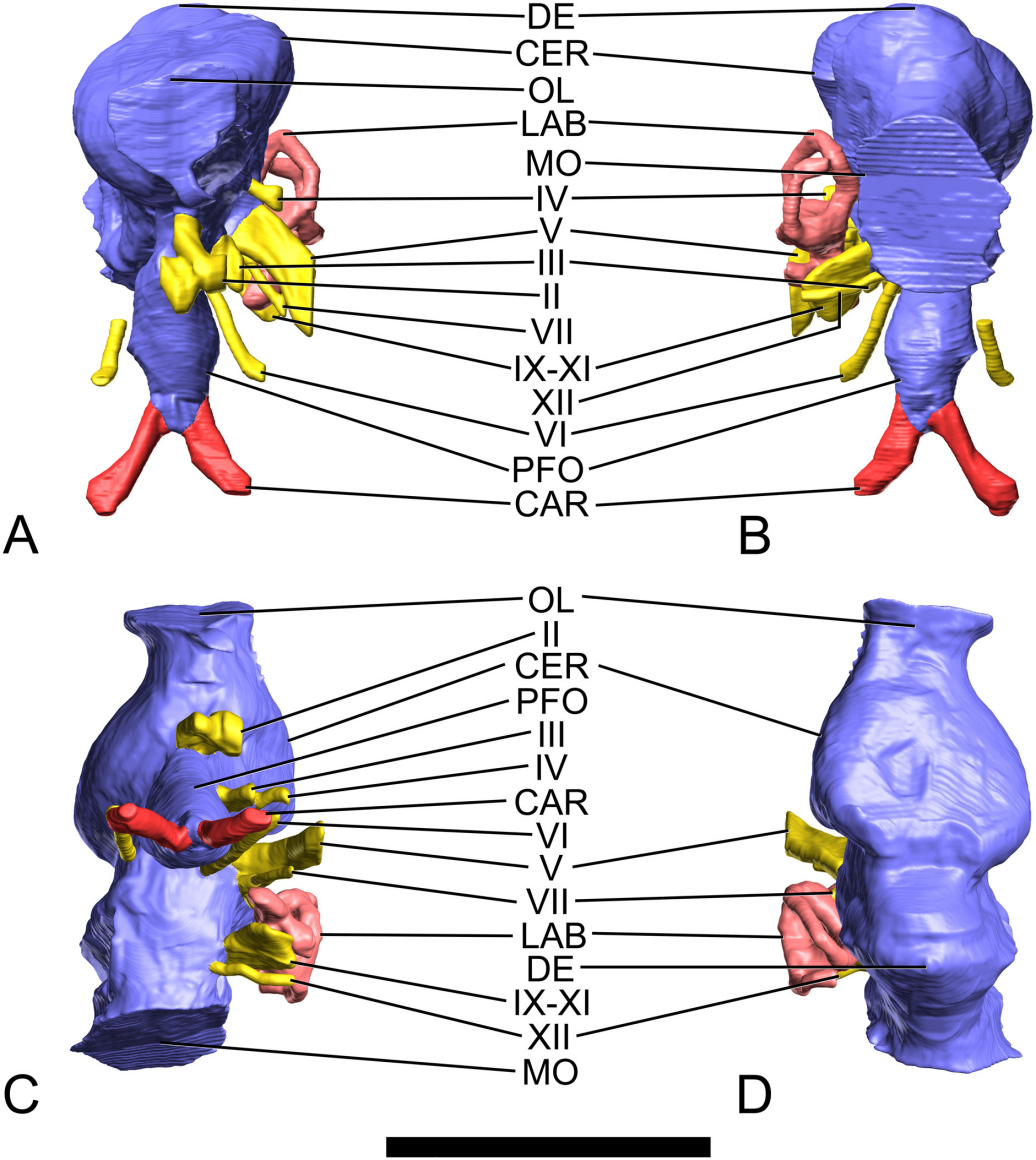
Surface-rendered CT-based images of the cranial endocast and endosseous labyrinth of the indeterminate titanosaurian sauropod braincase MCCM-HUE-1667 from the Cretaceous of Fuentes, Spain. In rostral (A), caudal (B), ventral (C), and dorsal (D) views. Dorsal is to the top in (A, B), rostral is to the top in (C, D). Abbreviations as in Fig 3. Scale bar equals 5 cm.

The frontal in MCCM-HUE-1667 inclines much more rostroventrally than the same bone in *Ampelosaurus atacis* ([18]: fig. 4.2) and *Ampelosaurus* sp. ([9]: figs 1, 2, S1, S2, S3). The frontal of *Ampelosaurus atacis* ([18]: fig. 4.2) is short and close in outline to that in MCCM-HUE-1667. However, the lateral margin of the frontal in *Ampelosaurus* sp. ([9]: figs 1, 2, S1, S2, S3) is embayed. In comparison with the frontal in FAM 03.064 ([12]: figs 2–5), the corresponding element in MCCM-HUE-1667 extends much less laterally. In FAM 03.064, the rostral border of the frontal is also slightly, but uniformly, convex, without any rostral projection. It is also flatter (less concave) ventrally. The frontal of the unnumbered MNHN braincase ([19]: fig. 2, as parietal) is poorly preserved on both sides, but appears to have borne a rostrolateral projection, whose initial expansion cannot be appraised. The frontal of FGGUB 1007 ([20]: fig. 15) is narrower than that of MCCM-HUE-1667. Its rostral portion is oriented steeply ventrally.

#### Parietal

Because of a lack of evidence for an interparietal suture, this bone is treated here as a single entity. The parietal is a rostrocaudally short, dorsoventrally shallow but transversally wide bone (Figs 2A, 2C, 3A and 3C, S1 Fig). It is characterized by paired crescentic crests, the transverse nuchal crests, on both sides of the median plane (Figs 2A and 3A, S1 Fig). These crests are concave-caudal to provide the insertion of the strong epaxial muscles. Joining at the caudal tip of the parietal, they form the dorsal part of the sagittal nuchal crest where they meet the supraoccipital (Figs 2A and 3A, S1 Fig). The lateral wings of the parietal join the proximal portion of the otoccipital rostrally (Figs 2A and 3A, S1 Fig). The convex margin of the transverse nuchal crest forms the caudal border of the supratemporal fenestra (Figs 2A and 3A, S1 Fig). There is no postparietal foramen (Figs 2A and 3A, S1 Fig). The parietal extends 30.6 mm rostrocaudally along the midline.

The parietal of *Ampelosaurus atacis* was only briefly described by Le Loeuff ([18]: 119–120, fig. 4.2) as thick and forming a “marked promontory” similar to that of *Antarctosaurus wichmannianus* ([21]: pl. 28). The parietal of MCCM-HUE-1667 is similar to *Antarctosaurus* in the form of the transverse nuchal crest ([21]: pl. 28 fig. 2; [22]: pls 63, 64 fig. e). The same resemblance is found between MCCM-HUE-1667 and *Ampelosaurus* sp. ([9]: figs 1, 2, S1, S2, S3) and, indeed, other taxa. The latter specimen, however, does not show the flat surface that is present at the top of the parietal in MCCM-HUE-1667 but instead a concavity. Regarding the depth of the caudal concavity formed by the transverse nuchal crest (nuchal fossa), the parietal in MCCM-HUE-1667 is closer in morphology to that in FAM 03.064 ([12]: figs 2, 5a) than to that of *Ampelosaurus atacis*. The parietal in MCCM-HUE-1667 is strikingly different from that in FGGUB 1007 ([20]: fig. 15). In the latter, it is proportionally larger, does not bear obvious transverse nuchal crests, and shows laterally, near its suture with the frontal, peculiar small and ovate protuberances.

#### Supraoccipital

The supraoccipital is small and rounded, forming, along with the parietals, a low sagittal nuchal crest (Figs 2A, 2E, 3A and 3F, S1 Fig). It contacts the parietal dorsally and the otoccipitals (exoccipitals) laterally (Figs 2A, 2E, 3A and 3F, S1 Fig). Ventrally, it participates in the rim of the foramen magnum, albeit in a very limited way (Figs 2E and 3F, S1 Fig). The supraoccipital is approximately 24.3 mm wide, 13.0 mm tall, and 20.2 mm long.

The supraoccipital has been lost or remains undescribed in most of the other European Late Cretaceous titanosaurian braincases. It is poorly preserved in *Ampelosaurus* sp. ([9]: figs 1, 2, S1, S2, S3), but was possibly not significantly different from that in MCCM-HUE-1667. The supraoccipital of the fragmentary braincase described by Le Loeuff et al. ([23]: pl. 1) is small and basically like that of MCCM-HUE-1667.

#### Otoccipital

This bone results from the early co-ossification of the exoccipital and opisthotic, which is a common feature of adult archosaurs and other diapsids (e.g., [24]: 7). The foramen magnum is dorsoventrally elongate (20.9x29.5 mm) and, as a result, it is bordered almost entirely by the otoccipitals, in that both the supraoccipital dorsally and the basioccipital ventrally contribute much less (Figs 2E and 3F, S1 Fig). The caudal surface of the otoccipital is flat (Figs 2E and 3F, S1 Fig). Rostrally, the otoccipital constitutes most of the caudal wall of the adductor chamber, except for the dorsalmost portion, which is constituted by the parietal (Figs 2A, 2D, 3A and 3C–3E, S1 Fig). The metotic foramen opens within the otoccipital close to the prootic, at the level of the occipital condyle, at the bottom of a cavity of the lateral wall of the braincase (auditory recess) that is roofed by the paroccipital process (Fig 3C and 3D, S1 Fig). As revealed by the CT scan data, the fenestra cochleae (= fenestra pseudorotundum, foramen perilymphaticum) opens into the rostral wall of metotic foramen, as is typically the case in sauropods. The oval window (fenestra ovalis = fenestra vestibuli) opens at the dorsal extremity of the depression, where it is bordered by the otoccipital and prootic; this area of the fossil itself is not particularly clear via visual observation, but these features are visible in the CT scan data (Fig 3D, S1 Fig). The paroccipital process is aliform, much taller dorsoventrally than long rostrocaudally. It strongly arches ventrally and curves medially back towards the braincase at it distal end (Figs 2E and 3F, S1 Fig). The dorsolateral notch in the paroccipital process for the posttemporal foramen is relatively broad and shallow (Figs 2E and 3F, S1 Fig). The base of the paroccipital process is embedded in matrix rostrally so that it is difficult to assess the configuration of the crista otosphenoidea (= crista prootica), but the CT scan suggests that the crest is comparable to that of other sauropods. There is a distinct crest on the rostral surface of the distal portion of the paroccipital process, which appears dorsally near the mid-line but merges with the medial border of the bone ventrally (Figs 2D and 3E, S1 Fig).

**Fig 6:**
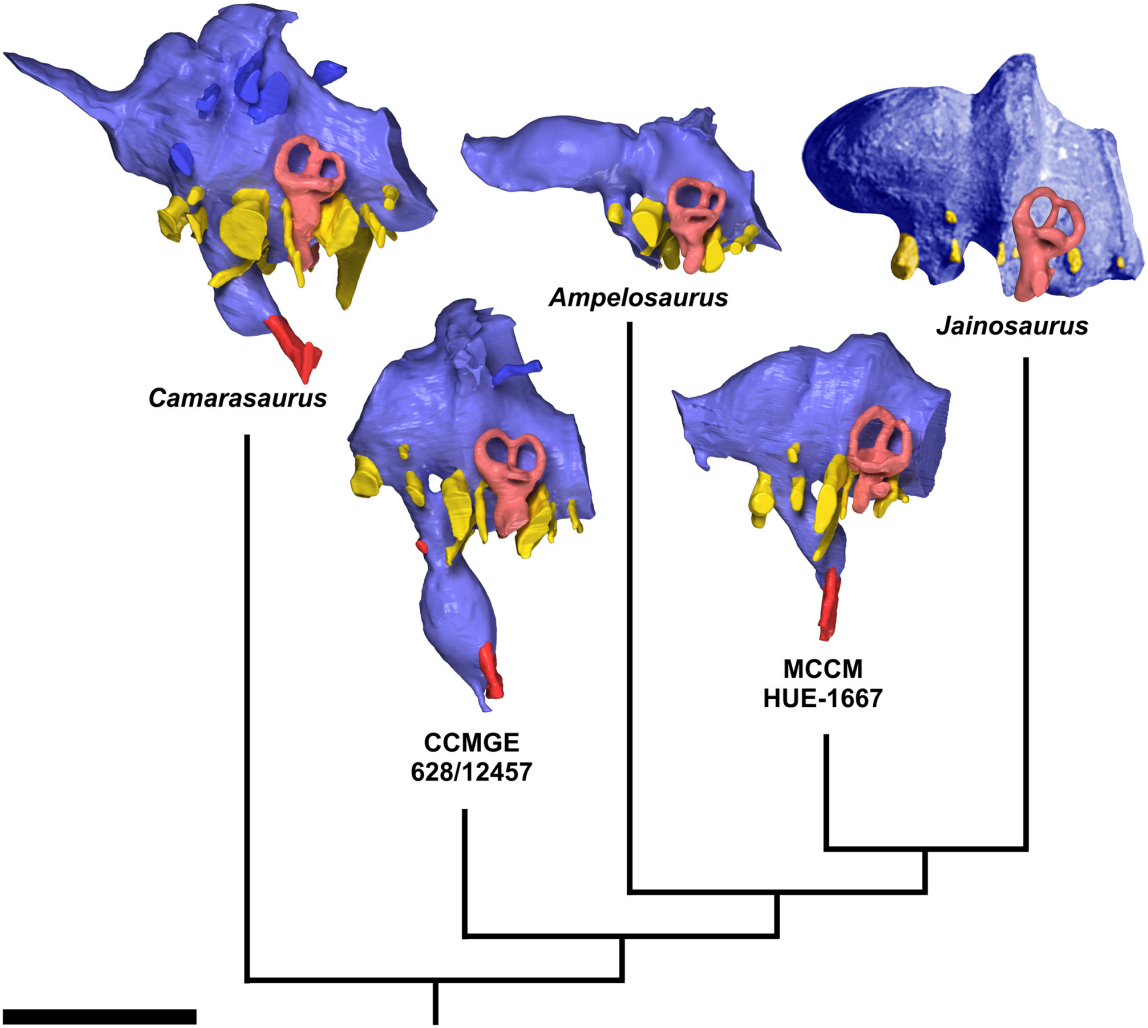
Endocasts of some sauropod taxa discussed in the text, displayed on a cladogram. From left: *Camarasaurus lentus* (CM 11338), indeterminate titanosaurian from Uzbekistan (CCMGE 628/12457), *Ampelosaurus* sp. from Spain (MCCM-HUE-8741, right side mirrored), indeterminate titanosaurian from Spain (MCCM-HUE-1667), and *Jainosaurus septentrionalis* (composite). In left lateral view. Dorsal is to the top. All but E digital endocasts; *Jainosaurus septentrionalis* is a montage of a photograph of the physical endocast (incomplete rostrally) made from the lectotypic braincase (GSI K27/497 ([28]: fig. 7A), right side mirrored and colorized) combined with the digital reconstruction of the endosseous labyrinth from a different specimen referred to the same species (ISI R162). CCMGE 628/12457 ([10]: fig. 3A, B) is depicted in a more basal phylogenetic position than either *Ampelosaurus* sp., MCCM-HUE-1667 or *Jainosaurus septentrionalis* based on its well-developed “middle cerebral vein” system. Scale bar equals 5 cm.

In contrast with the condition in MCCM-HUE-1667, the foramen magnum of *Ampelosaurus atacis* ([18]: fig. 4.2) and *Ampelosaurus* sp. ([9]: figs 1, 2, S1, S2, S3) is not dorsoventrally elongate. In these taxa, the paroccipital process is not vertical and hanging laterally as in MCCM-HUE-1667, but rather is more dorsoventrally oblique. It is also oriented laterally in caudal view, at least proximally, whereas that in MCCM-HUE-1667 arches strongly ventrally from the proximal portion (Figs 2E and 3F, S1 Fig), as is often the case in various Gondwanan titanosaurians such as *Saltasaurus loricatus* ([22]: pl. 19, 47 fig. 2a, b; [25]: fig. 6C). The foramen magnum shows a similar outline in MCCM-HUE-1667 and the specimen described by Le Loeuff et al. ([23]: pl. 1). The paroccipital process in the latter specimen seems to have been originally arched ventrally as in MCCM-HUE-1667. However, Le Loeuff et al. [23] noted a small protuberance on the exoccipital (Le Loeuff et al. [23] believed they could perceive the limit between the exoccipital and the opisthotic) near the foramen magnum. This protuberance was compared with that seen on the Dongargaon sauropod braincase ([26]: fig. 2) and correctly interpreted similarly as an articular facet for the proatlas (the specimen from India is now referred to as of the *Isisaurus* morph [27,28]). Two small depressions lateral to the protuberance were also noted. No such protuberance and depressions associated with the proatlas are noticeable on the otoccipital of MCCM-HUE-1667 (Figs 2E and 3F, S1 Fig). The paroccipital process in the unnumbered MNHN partial braincase ([19]: fig. 2, pls 5–6) is not as strongly pendant and vertically oriented as in MCCM-HUE-1667. Admittedly, both specimens exhibit some deformation but not to a sufficient extent to account for the difference in the two specimens. The paroccipital process of the unnumbered MNHN specimen also dilates distally, whereas that of MCCM-HUE-1667 tapers distally (Figs 2D, 2E, 3E and 3F, S1 Fig).

#### Basioccipital

The basioccipital is a relatively dorsoventrally tall but rostrocaudally short bone (Figs 2E and 3F, S1 Fig). It probably constituted most of the occipital condyle (Figs 2E and 3F, S1 Fig). The latter has been damaged through excavation work, although it was likely hemispheric in morphology and transversally wider than the foramen magnum, as seen in a variety of sauropods. A subcondylar recess separates the occipital condyle from each basal tuber, which progressively arises with an irregular outline from the ventrolateral border of the caudal surface of the basioccipital (Figs 2E and 3F, S1 Fig). The basal tubera are relatively close to one another (Figs 2E and 3F, S1 Fig), which is consistent with the configuration in most sauropods, in which the basal tubera are close to one another and best seen in a caudal view of the braincase (*Spinophorosaurus nigerensis* is a notable exception to this pattern; [29]: figs 1–3, S1, S2, S3). The basioccipital is perforated caudolaterally by the hypoglossal canal (Fig 3D, S1 Fig). The bone is 55.3 mm tall in the midline and 52.8 mm wide at the level of the basal tubera.

Whereas the occipital condyle was probably knob-like in MCCM-HUE-1667, that of *Ampelosaurus atacis* ([18]: fig. 4.2) and *Ampelosaurus* sp. ([9]: figs 1, 2, S1, S2, S3) may have been distinctly wider transversely than dorsoventrally tall. Although it cannot be confirmed, the entire basioccipital of *Ampelosaurus atacis* and *Ampelosaurus* sp. may have been less tall dorsoventrally than that in MCCM-HUE-1667. In *Lirainosaurus astibiae* ([30]: figs 2–4), the basioccipital forms a generally hemispheric occipital condyle with a short neck, as was possibly the case in MCCM-HUE-1667. However, in that taxon the surface of the basioccipital between the occipital condyle and the basal tubera is not excavated as in MCCM-HUE-1667 and lacks the subcondylar recess or foramen. In *Lirainosaurus astibiae*, the basal tubera are rounded and prominent and, therefore, unlike those in MCCM-HUE-1667. Díez Díaz et al. [30] noted that the distal surface of each basal tuber is pierced by a foramen and regarded this configuration as autapomorphic of *Lirainosaurus astibiae*. Nevertheless, it is probably homologous to the notch observed at this place in *Jainosaurus septentrionalis* ([28]: fig. 6; [31]: fig. 11). In the unnumbered MNHN braincase ([19]: fig. 2, pls 5–6), the occipital condyle appears to have had a relatively longer neck than that in MCCM-HUE-1667. Similarly, the basal tubera are much more rounded and prominent caudally in the unnumbered MNHN braincase than in MCCM-HUE-1667 and the zone of the basioccipital between the occipital condyle and the basal tubera is more excavated in the former specimen than in the latter.

#### Parabasisphenoid

As is generally the case in sauropods, as well as in most dinosaurs and crocodilians and indeed many other reptiles [32], the basisphenoid and parasphenoid are indistinguishably fused with each other (at least in adults). They are, therefore, described here as a single unit, the parabasisphenoid. It forms the ventral extremity of the braincase, just beneath the basal tubera (Figs 2E and 3F, S1 Fig). Both basipterygoid processes are broken near their base (Figs 2D, 2E, 3E and 3F, S1 Fig). However, it can be inferred from what remain that they were of light build, largely square in proximal cross-section and directed essentially ventrally as they emerged from the basicranium (approximately 45° to skull roof). They probably diverged from one another in a widening U-shaped fashion (approximately 45°). Ventrally, the parabasisphenoid forms a triangular concavity that points rostrally (Figs 2B and 3B, S1 Fig). Only the very base of the parasphenoidal rostrum (cultriform process) is preserved (Figs 2B–2D and 3B–3E, S1 Fig), suggesting that it was, as in other sauropods, a delicate, blade-shaped structure. Lateral to this base of the parasphenoidal rostrum, the parabasisphenoid is slightly concave. The abducens canal opens on the dorsalmost extension of the rostral surface of the parabasisphenoid, very close to the ventral limits of the laterosphenoid, and about equidistant from the ventral midline and lateral edge of the braincase. Neither of the two external openings of the internal carotid artery’s cerebral branch canals are visible in the fossil specimen, but the CT scan data shows that they open lateral to the base of the parasphenoidal rostrum on the rostral surface of the parabasisphenoid (Fig 3D, S1 Fig). As preserved, the parabasisphenoid is only 13.4 mm long and 40.9 mm wide at the level of the base of the basipterygoid processes.

**Fig 7:**
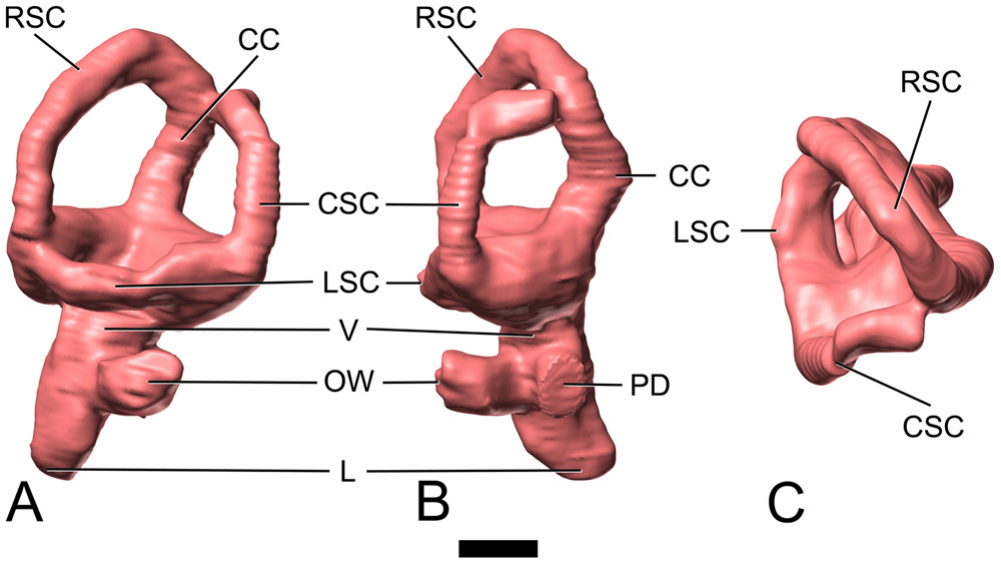
Surface-rendered CT-based images of the left endosseous labyrinth of the indeterminate titanosaurian sauropod braincase MCCM-HUE-1667 from the Cretaceous of Fuentes, Spain. In lateral (A), caudal (B), and dorsal (C) views. Dorsal is to the top. Abbreviations: CC, common crus; CSC, caudal semicircular canal; L, lagena; LSC, lateral semicircular canal; OW, oval window; PD, perilymphatic duct; RSC, rostral semicircular canal; V, vestibule. Scale bar equals 5 mm.

**Fig 8:**
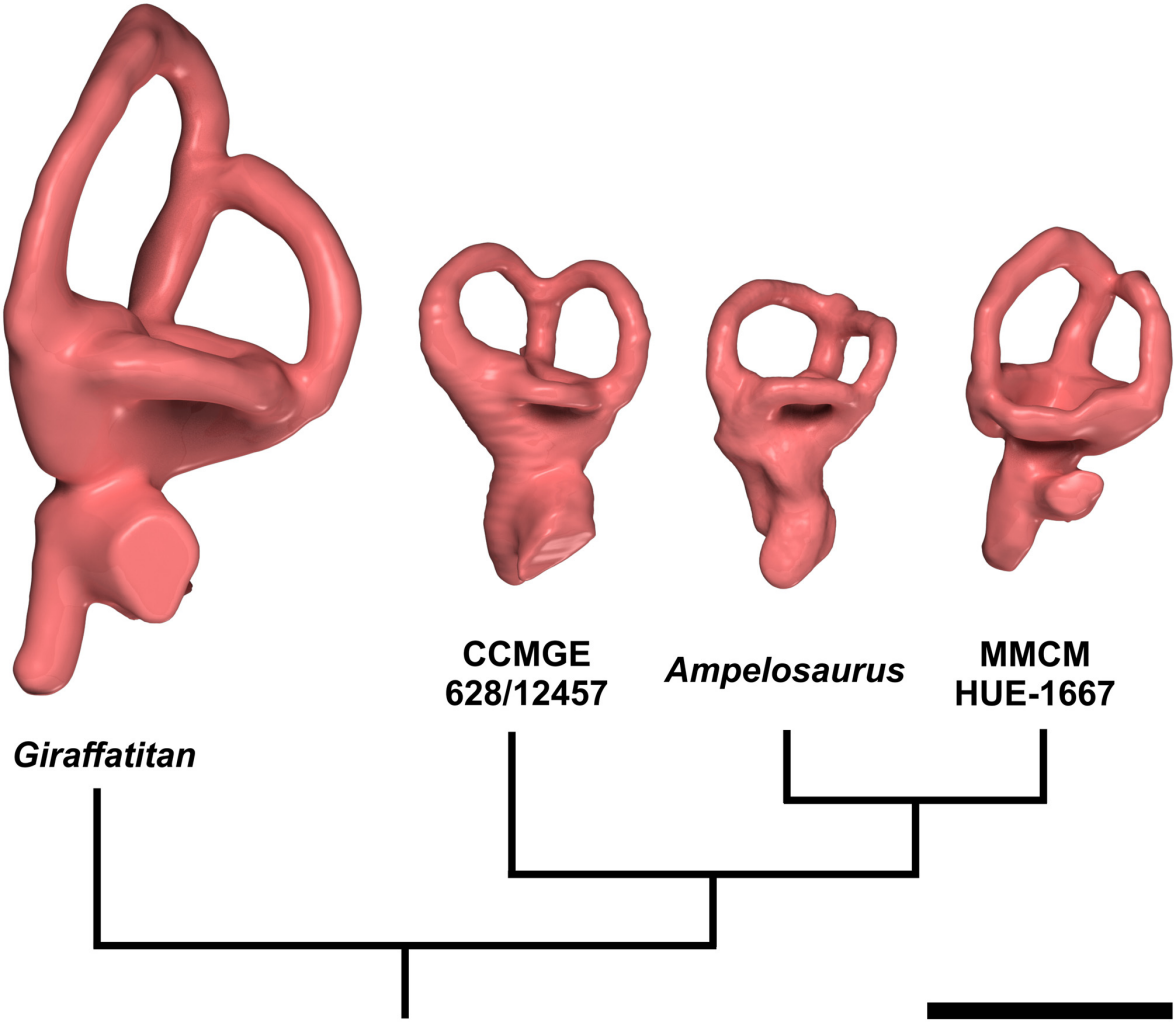
Endosseous labyrinths of some sauropod taxa discussed in the text, displayed on a cladogram. In lateral view. Dorsal is to the top. From left: surface renderings of CT images of the inner ears of *Giraffatitan brancai* (MB.R.2180.22.1–4, right side mirrored), *Ampelosaurus* sp. from Spain (MCCM-HUE-8741, right side mirrored), the indeterminate titanosaurian from Spain (MCCM-HUE-1667, left side), and the indeterminate titanosaurian from Uzbekistan (CCMGE 628/12457, left side). Scale bar equals 20 mm.

The body of the parabasisphenoid in MCCM-HUE-1667 is relatively much shorter rostrocaudally than that of *Lirainosaurus astibiae* ([30]: figs 3, 4C, 6, 7B, 8B). Based on their proximal sections, the basipterygoid processes were possibly more robust in *Lirainosaurus astibiae* than in MCCM-HUE-1667. The parabasisphenoid of *Lirainosaurus astibiae* is pierced by well-characterized foramina for the entrance of the cerebral branch of the right and left internal carotid arteries ventrally and the exit of the right and left abducens nerve (cranial nerve [CN] VI) rostrally. The preservational state of MCCM-HUE-1667 obscures the location of these foramina externally, but their positions can be determined by the internal canals visible in the CT scan data (Fig 3D, S1 Fig). In the poorly preserved braincase described by Le Loeuff et al. [23], these authors assumed the presence of a strong basipterygoid process, but this is actually a remnant of the right basal tuber: nothing from the basisphenoid and parasphenoid seems to have been preserved in that specimen. The parabasisphenoid of the unnumbered MNHN specimen ([19]: fig. 2, pls 5–6) does not show the well-defined triangular concavity visible on the ventral surface of this bone complex in MCCM-HUE-1667. Comparisons of the parabasisphenoid of MCCM-HUE-1667 with that of FAM 03.064 ([12]: figs 3–5) are made difficult by the incomplete preservation of the latter specimen ventrally. In the peculiar braincase reported by Weishampel et al. ([20]: fig. 15), the basisphenoid and parasphenoid form a much rostrocaudally longer complex than in MCCM-HUE-1667. In comparison with the braincases of titanosaurians illustrated by Curry Rogers and Wilson [25: fig. 6] in caudal views, the subcondylar region of the basicranium in MCCM-HUE-1667 most closely resembles that of *Jainosaurus septentrionalis* in both its overall relative proportions and the shape of the basal tubera.

#### Prootic

The prootic is largely concealed because the adductor chamber is partially filled with matrix on both sides. The chamber is very short rostrocaudally (as is the supratemporal fenestra) and the prootic is even shorter. It inserts as a wedge between the otoccipital caudally and the laterosphenoid rostrally and it is limited rostrodorsally by the frontal. The left side shows that the ventral vertex of the prootic is pierced by the large trigeminal foramen (for CN V), which is caudally bordered by the crista otosphenoidea (Fig 3C and 3D, S1 Fig). The singular foramen indicates an extracranial position of the trigeminal nerve, as in most sauropods (see e.g., [33]). Its outline is somewhat like a triangle with convex sides pointing ventrally (Fig 3C and 3D, S1 Fig); it is 15.3 mm tall dorsoventrally and 9.7 mm long rostrocaudally. The facial foramen (for CN VII) is visible on the left side (Fig 3C and 3D, S1 Fig). It opens immediately caudal to the crista otosphenoidea, approximately at the level of the ventral tip of the trigeminal foramen. It is shaped like a dorsoventrally elongate ellipsoid (9.7 mm tall dorsoventrally and 4.3 mm long rostrocaudally).

The prootic of MCCM-HUE-1667 is relatively rostrocaudally shorter than that of *Ampelosaurus atacis* ([18]: fig. 4.2), *Ampelosaurus* sp. ([9]: figs 1, 2, S1, S2, S3), and FGGUB 1007 ([20]: fig. 15). No such difference is discerned with the prootic of FAM 03.064 ([12]: figs 4, 5E-F) and, possibly, that of *Lirainosaurus astibiae* ([30]: figs 3–4), which are both short as well.

#### Laterosphenoid

The laterosphenoid is a rostrocaudally short bone that is bounded caudally by the prootic, rostroventrally by the orbitosphenoid, and dorsally by the frontal (Figs 2B–2D and 3B–3E, S1 Fig). It extends laterally into a flat, short, digitiform capitate process (Figs 2B–2D and 3B–3E, S1 Fig). The ventral border of this process forms a crest (crista antotica) that continues ventrally toward the parabasisphenoid (Figs 2B–2D and 3C–3E, S1 Fig). In between the latter bone and the capitate process, it constitutes the rostral limit of the trigeminal foramen (Fig 3C and 3D, S1 Fig). At the level of the trigeminal foramen, the laterosphenoid extends rostrocaudally only 7.1 mm, but it is about 40 mm tall.

The laterosphenoid of MCCM-HUE-1667 appears similar to that of *Ampelosaurus* sp. ([9]: figs 1, 2, S1, S2, S3). Nonetheless, the slight rostral curvature of the laterosphenoid of *Ampelosaurus* sp. cannot be confirmed in MCCM-HUE-1667 due to preservation issues.

#### Orbitosphenoid

The orbitosphenoids have fused completely into a single, median unit, which constitutes the rostroventral extremity of the braincase (Figs 2B–2D and 3B–3E, S1 Fig). This element forms an acute ventral carina (Figs 2B, 2D, 3B and 3E, S1 Fig). As a result, it presents a short and wide Y-shape in transverse section. The carina is in line with the base of the parasphenoidal rostrum described above (Figs 2D and 3E, S1 Fig), and indeed the cartilaginous interorbital septum attached to both these structures. The orbitosphenoids form the floor of the olfactory aperture (Figs 2D and 3E, S1 Fig), which is roofed by a rostral rising of the frontals, and enclose the laterally directed optic foramina (Fig 3D, S1 Fig). They also border rostrally the oculomotor and trochlear foramina (for CN III and CN IV, respectively), which are oval and slit-like, respectively (Fig 3D, S1 Fig). The average diameter of the ocumolotor foramen (5.4 mm) is in between that of the relatively large optic aperture and that of the diminutive trochlear opening (Fig 3D, S1 Fig). The united orbitosphenoids are 31.7 mm wide transversely at the middle part and about 24 mm tall dorsoventrally. Comparisons of the orbitosphenoid of MCCM-HUE-1667 with that of other European titanosaurians is especially difficult as this bone is often poorly preserved or even lacking altogether.

### Neuroanatomy

Although Marsh ([34]; see also [35]: pl. 8; [36]: fig. 34) initiated the paleoneurological study of sauropod dinosaurs, it was not until more than three decades later that a physical endocast made from a specimen of this group was figured in detail ([37]: fig. 16A). The first paleoneurological data on titanosaurian sauropods were provided by Huene ([21]: pl. 28) from the braincase foramen pattern of *Antarctosaurus wichmannianus*. A few years later, Huene and Matley ([38]: fig. 6) figured for the first time a “braincast” of a titanosaurian, “*Antarctosaurus*” *septentrionalis*. In the present case, the CT scan data permitted a comprehensive rendering of the cranial endocast and endosseous labyrinth (Figs 4–7, S1 Fig). The completeness of the braincase allowed reconstructing the course of every cranial nerve of the left side as they traverse the braincase wall, which has not been possible so far in most sauropod taxa. Below, we refer to the reconstructed digital casts of bone-bounded spaces that housed soft-tissue structures as if they were the structures themselves (e.g., “trigeminal nerve” rather than “digital cast of trigeminal foramen”).

#### Brain endocast

The olfactory tract is extremely short (Figs 4, 5C and 5D, S1 Fig), which is related to the small extent of the frontal rostrocaudally (Fig 3D, S1 Fig). As far as it can be observed, the olfactory bulb is also short (Figs 4, 5C and 5D, S1 Fig). There is little doubt that the olfactory region of the nasal cavity, where the olfactory nerve (CN I) originated, was situated very caudally. This is consistent with shortened (or highly modified) nasal bones and, therefore, greatly retracted or broadened external nares as in other sauropods [39]. The same configuration of the olfactory lobe is observed in other titanosaurians ([8]: figs 1–6; [9]: figs 3, S1, S2, S3; Fig 6).

The longitudinal axis of the olfactory tracts is almost in line with the medulla oblongata (Fig 4, S1 Fig). This stands in sharp contrast with the condition in the basal titanosauriform *Giraffatitan brancai*, in which the pontine and cerebral flexures of about 50° each result in an endocast that appears sigmoid or “flexed” in lateral view ([40]: figs 1, 2A, B). An endocast that is sigmoid to varying degrees in lateral view is, in fact, widely distributed in non-titanosaurian sauropods (e.g., *Shunosaurus lii* ([41]: fig. 11); *Diplodocus longus* ([42]: fig. 6.9)), which suggests that a straighter (less sigmoidal or “flexed”) morphology, as seen in MCCM-HUE-1667, is the derived state. The total volume of the endocast is 41.63 cm^3^, over 6% of which is devoted to the pituitary fossa.

The cerebral region is well delimited and constitutes the widest portion of the endocast (Fig 5D, S1 Fig). It is separated from the midbrain-hindbrain complex through a marked constriction (Figs 4, 5C and 5D, S1 Fig), which is caused by the laterosphenoid pillar. A second medial narrowing situated more caudally, but of similar morphology and nearly as extensive, makes room for the otic capsule (Figs 4, 5C and 5D, S1 Fig). The presence of the cerebellum is not reflected on the endocranial surface, and there is no fossa auriculae cerebelli (which in life housed the cerebellar flocculus) visible, whereas a small flocculus is present on the endocast of *Giraffatitan brancai* ([40]: figs 1, 2A-D). The prominence of the flocculus on the endocast varies greatly within Sauropoda. Thus, it is well defined in cf. *Cetiosaurus oxoniensis* ([33]: fig. 6) and a few other basal sauropodomorphs (e.g., *Massospondylus carinatus* ([43]: fig. 1G]), but is essentially absent from the endocasts in all studied titanosaurians from Europe, South America, and India, as well as most diplodocoids, *Camarasaurus*, and some others (see e.g., [42]: figs 6.8, 6.9; Fig 6). In contrast with *Giraffatitan brancai* ([40]: figs 1, 2A, B) and *Camarasaurus lentus* ([42]: fig. 6.8A, B; Fig 6), the forebrain of MCCM-HUE-1667 was not covered by a large dural venous sinus (Figs 4, 5A and 5D, S1 Fig), but rather is more similar to the endocast of *Jainosaurus septentrionalis* ([28]: fig. 7A; Fig 6), *Bonatitan reigi* ([8]: figs 2, 3), and *Antarctosaurus wichmannianus* ([8]: fig. 5) in largely lacking a rostral dural expansion. However, the hindbrain has a large caudal dural expansion above the cerebellar region (Figs 4, 5A, 5B and 5D, S1 Fig), which may be due to the presence of an enlarged torcular herophili, which is a confluence between the transverse (“middle cerebral vein”), dorsal sagittal, and occipital sinuses. In contrast, *Ampelosaurus* sp. does not support any voluminous caudal dural expansion, but only a small winged protrusion ([9]: figs 3A-C, S1, S2, S3; Fig 6). In comparison with *Ampelosaurus* sp. ([9]: figs 3, S1, S2, S3), and taking the lateral semicircular canal as reference for the alert posture of the head [42], the dorsal surface of the forebrain is much less horizontal in MCCM-HUE-1667 (Fig 6). The latter specimen also shows a much more abrupt dorsal inflection between the olfactory tracts and the more caudal part of the forebrain in lateral view (Fig 6) and much less marked medial constrictions in dorsal view.

As far as the overall morphology of the endocast is concerned, MCCM-HUE-1667 is much closer to *Jainosaurus septentrionalis* ([28]: fig. 7) than to *Ampelosaurus* sp. (Fig 6). MCCM-HUE-1667 is also reminiscent of *Bonatitan reigi*, but the latter presents a more pronounced “collar” around the transition between the forebrain and the midbrain-hindbrain ([8]: figs 2, 3; [44]: fig. 13.6, 13.7). In dorsal view, the caudal border of the forebrain is straight in *Bonatitan reigi* ([8]: fig. 2B; [44]: fig. 13.6), whereas it is fairly curved in MCCM-HUE-1667 (Fig 5D, S1 Fig). The same difference stands between MCCM-HUE-1667 on one side and *Antarctosaurus wichmannianus* and MGPIFD-GR 118 (an indeterminate titanosaurian from Argentina) on the other ([8]: figs 5B, 6B). The endocast of MCCM-HUE-1667 resembles more closely that of MGPIFD-GR 118 than that of either *Bonatitan reigi* or *Antarctosaurus wichmannianus*, but MGPIFD-GR 118 presents a unmistakably steeper dorsocaudal edge in lateral view ([8]: fig. 6A). Indeed, *Bonatitan reigi*, *Antarctosaurus wichmannianus*, as much as MGPIFD-GR 118 appear more sigmoid in lateral view than MCCM-HUE-1667 ([8]: figs 2A, 5A, 6A). Moreover, the caudal dural expansion of *Antarctosaurus wichmannianus* does not extend appreciably more dorsally than the forebrain in contrast with the condition in MCCM-HUE-1667 ([8]: fig. 5A). On the whole, *Antarctosaurus wichmannianus* presents a somewhat more compact morphology than MCCM-HUE-1667 ([8]: fig. 5).

As is usual in sauropods [42,45], the endocast is set apart by a large hypophysis (pituitary) that extends ventrally (Figs 4, 5A and 5B, S1 Fig). Similar to *Giraffatitan* ([40]: figs 1, 2A, B), but in contrast with most other sauropods (see e.g., [42]: figs 6.8, 6.9; [46]: fig. 10; Fig 6), the infundibulum is not constricted (Fig 4, S1 Fig). The entire hypophysis, which is most inflated at about 3/5 of its length from its junction with the ventral surface of the brain, does not vary considerably in diameter along the proximal-to-distal axis (Fig 4, S1 Fig). The caudoventral orientation of the hypophysis is attained through a curvature of the infundibular stalk (Fig 4, S1 Fig). The angle between the lateral semicircular canal and the hypophysis is about 45° (Fig 4, S1 Fig), which is more acute than in *Ampelosaurus* sp. (~55°; [9]: figs S1, S2, S3; Fig 6), *Bonatitan reigi* (~65°; [8]: fig. 2A), *Antarctosaurus wichmannianus* (~75°; [8]: fig. 5A), and MGPIFD-GR 118 (~85°; [8]: fig. 6A).

#### Cranial nerves

The optic nerve (CN II) is short but stout (Figs 4 and 5A, S1 Fig). Each leaves the braincase through its own aperture (Fig 3D, S1 Fig). It compares well in most respect to the conformation in the South American taxa examined by Paulina Carabajal ([8]: figs 2A, 3, 5A, 6A, C, 8B). As in most titanosaurians, the optic nerve canal is directed almost directly laterally (Figs 4, 5A and 5C, S1 Fig) rather than rostrolaterally as in most other dinosaurs and more basal sauropodomorphs (see e.g., [42]), presumably reflecting the retraction and telescoping of the structures caudal to the expanded narial region, rotating the orbits far laterally and compressing the adductor chamber.

The oculomotor nerve (CN III) is ellipsoid in cross section (Fig 4, S1 Fig). It courses ventrolaterally over a short distance within the braincase wall before entering the orbit (Fig 3D, S1 Fig). It emerges laterally on the infundibulum (pituitary stalk) as in *Antarctosaurus wichmannianus* ([8]: fig. 5A) and *Ampelosaurus* sp. ([9]: figs 3A, S1, S2, S3; Fig 6), but more rostrally than in *Jainosaurus septentrionalis* ([28]: fig. 7; Fig 6), *Bonatitan reigi* ([8]: figs 2A, 3), *Saltasaurus loricatus* ([8]: fig. 8B), and MGPIFD-GR 118 ([8]: fig. 6A).

The trochlear nerve (CN IV) is tiny and slit-like in cross section at its distal extremity (Fig 4, S1 Fig). Its short route is mainly laterally (Figs 4, 5A and 5C, S1 Fig). It is dorsal to the mid-infundibulum (Fig 4, S1 Fig), as in *Antarctosaurus wichmannianus* ([8]: fig. 5A), but more rostral than in *Jainosaurus septentrionalis* ([28]: fig. 7; Fig 6), *Bonatitan reigi* ([8]: figs 2A, 3), *Saltasaurus loricatus* ([8]: fig. 8B), and MGPIFD-GR 118 ([8]: fig. 6A).

The trigeminal nerve (CN V) is the first cranial nerve caudal to the dorsum sellae (Figs 3 and 4, S1 Fig). It emerges from the widest portion of the hindbrain. It is the largest of the cranial nerves, as usual in archosaurs and other animals. The upside-down cordiform outline in proximal cross section is related to the *in vivo* division of this nerve into rostral (ophthalmic, CN V_1_) and caudal (maxillomandibular, CN V_2,3_) rami. The trigeminal nerve is oriented mostly ventrolaterally (Figs 4, 5A, 5C and 5D, S1 Fig). The position and configuration of the cranial nerve in MCCM-HUE-1667 is not significantly different from the condition in other taxa such as *Bonatitan reigi* ([8]: figs 1–3; [44]: fig. 13.4, 13.6, 13.7), although in the latter the nerve is directed mostly laterally.

The abducens nerve (CN VI) emerges ventrally from the brainstem, ventral to the trigeminal nerve (Figs 4 and 5C, S1 Fig). The root of this nerve is about similarly situated in MGPIFD-GR 118 ([8]: fig. 6), but it is much more dorsal in *Bonatitan reigi* ([8]: fig. 3). In contrast to most archosaurs, but as in other somphospondylans [8,9,47] (Fig 6), *Europasaurus* (unpub. data) and, homoplasiously, most coelurosaurian theropods [48,49], the abducens nerve passes lateral to the pituitary fossa rather than through it (Figs 4, 5A, 5B and 5D, S1 Fig). In MCCM-HUE-1667, the course of the abducens does not brush past the pituitary fossa as it does in *Ampelosaurus* sp. ([9]: figs 3, S1, S2, S3). The more medial path seen in the latter is closer to the plesiomorphic condition in which the nerve penetrates the pituitary fossa (see e.g., the basal titanosauriform *Giraffatitan brancai* ([40]: fig. 1)).

The facial nerve (CN VII) emerges from the brainstem immediately caudal to the trigeminal nerve (Figs 4 and 5C, S1 Fig) in close association with the vestibulocochlear nerve (CN VIII), as in other amniotes. The facial nerve runs alongside the trigeminal nerve and leaves the braincase just caudally to it, passing through a canal located fully within the prootic on the caudal side of the crista otosphenoidea (Fig 3D, S1 Fig).

As its name implies, the vestibulocochlear nerve (CN VIII) has two branches. The vestibular branch enters the vestibular labyrinth in the region of the ampulla of the rostral semicircular canal, which is typical in extant diapsids, as well. The more ventral cochlear branch could not be traced in the CT scan data, but is assumed to have been present.

The glossopharyngeal and vagoaccesory nerves (CN IX-XI) are combined together with other structures into a slightly oblique, dorsoventrally elongated, and remarkably slender metotic canal (Figs 4, 5B and 5C, S1 Fig). As noted above, the perilymphatic duct opens from the labyrinth into the rostral wall of the metotic canal. Therefore, the course of the glossopharyngeal nerve (CN IX), as in most other titanosaurians ([8]: figs 2, 3, 5, 6; [28]: fig. 7), does not run through a separate aperture. *Ampelosaurus* sp. ([9]: figs S1, S2, S3) has a slightly altered arrangement in which the glossopharyngeal nerve appears to have diverged from the rest of the contents of the metotic canal at its the lateral terminus. A narrow canal caudal to the metotic foramen in a fragmentary braincase from Argentina of presumed titanosaurian affinity (MPCA-PV-80) was identified by Paulina Carabajal ([8]: fig. 7A) to be an independent accessory nerve (CN XI). However, in sauropsids the accessory nerve combines intimately with the vagus nerve (CN X) and, indeed, it has long been remarked that in modern archosaurs these two nerves leaves the cranial cavity as one ([50]: 45). The course of this canal is parallel to a hypoglossal nerve (XII) and we speculate it could actually be a reduced rostral branch of the hypoglossal.

The hypoglossal nerve (CN XII) has a single root (Figs 4, 5B and 5C, S1 Fig) as in many other titanosaurians [8,9], with the exception of MPCA-PV-80 ([8]: fig. 7A), *Pitekunsaurus macayai* ([8]: 2150), *Rapetosaurus krausei* ([3]: 135), and CCMGE 628/12457 (an indeterminate titanosaurian from Uzbekistan; [10]: fig. 3A-D). Paulina Carabajal et al. ([51]: fig. 4A, B) also identified only one hypoglossal root in the dicraeosaurid diplodocoid *Amargasaurus cazaui*, but we conservatively reinterpret the duct infilling they designed as an accessory nerve as a rostral root of the hypoglossal. The hypoglossal nerve of MCCM-HUE-1667 may be homologous with the most caudal of the hypoglossal roots of non-titanosaurian sauropods, which is usually the largest (see [42]: figs 6.8C, 6.9C), although it is also very possible that there was no real variation in the actual nerve roots in life, but rather only variation in whether those nerve branches passed through one or two bony canals. The single canal in MCCM-HUE-1667 emerges from the medulla oblongata and extends laterally and slightly ventrally and caudally (Figs 4, 5B and 5C, S1 Fig). In contrast with other titanosaurians ([8]: figs 2, 3, 5, 6; [9]: figs 3, S1, S2, S3; [28]: fig. 7; Fig 6), the root of the hypoglossal nerve is very close to the metotic group in MCCM-HUE-1667. We suggest that this may be an autapomorphic feature.

#### Endocranial vasculature

The cerebral carotid arteries (which enter the ventral end of the pituitary fossa remarkably close to one another) are by far the main vascular structures of the endocast (Figs 4, 5A and 5B, S1 Fig). However, CT data also reveal the existence of a few small veins draining the forebrain that course through the braincase wall, but were not reconstructed. One of these leaves the neurocranium on the laterosphenoid-orbitosphenoid suture. Although more gracile, it is clearly homologous to the orbitocerebral vein that is present in some sauropods like *Diplodocus longus* ([42]: fig. 6.9) and *Camarasaurus lentus* ([42]: fig. 6.8). In addition, two very delicate veins appear to run ventrolaterally from the endocast on the laterosphenoid-frontal interface or approximately so. Whether they are diploic veins, as in *Diplodocus longus* ([42]: fig. 6.9A, D, F), or not is unclear. It is unquestionable that the main venous drainages run off through apertures shared with nervous structures (e.g., foramen magnum, trigeminal foramen). In *Bonatitan reigi*, a “middle cerebral vein”, which extends essentially dorsolaterally, is present well dorsal to the trigeminal nerve ([8]: figs 2A, 3; [44]: fig. 13.7). In the latter taxon, a canal of probably venous origin also courses through the dorsum sellae ([8]: figs 3). These two vascular structures, which recall the primitive sauropod *Spinophorosaurus nigerensis* ([29]: figs S1, S2, S3), might indicate that *Bonatitan reigi* occupies a more basal position than MCCM-HUE-1667 within the phylogenetic tree of titanosaurians. This is reinforced by the fact that the non-titanosaurian titanosauriform *Tambatitanis* appears to have had a well-developed dorsal-head/caudal-middle-cerebral vein system ([47]: pp. 11, 15, 17, fig. 4F). Very recently, Salgado et al. ([52]: fig. 14) indeed recovered *Bonatitan* in a relatively primitive position within titanosaurians.

#### Inner ear

The left endosseous labyrinth could be reconstructed (Fig 7, S1 Fig). The step-like transition between the dorsal two-thirds and the remaining ventral portion (Fig 7A and 7B, S1 Fig) probably reflects the functional separation of the inner ear into two main regions (vestibular system and lagena), as discussed by Witmer et al. ([42]: p. 80) in *Diplodocus*, *Camarasaurus*, and other sauropods. In fact, the vestibule-lagena transition should be approximately coincident with the tangent line that passes through the dorsal top of the oval window. Thus, the length of the lagena is 10.7 mm.

The vestibular system of the inner ear is somewhat contracted (i.e. the radius of the semicircular canals is small), but less so than in other titanosaurians (Fig 8), such as *Bonatitan reigi* ([8]: fig. 9A, E; [44]: fig. 13.8, 13.11), *Ampelosaurus* sp. ([9]: figs 4, S1, S2, S3), and CCMGE 628/12457 ([10]: fig. 4A-C). Compared with the situation in *Giraffatitan brancai* ([29]: fig. 6; [40]: fig. 3), the rostral semicircular canal of all titanosaurians, including CCMGE 628/12457 ([10]: fig. 4A-C), is much shorter (Fig 8). Although our knowledge of the morphology of the vestibular system among sauropod taxa is still deficient, this equalization of the length of the two vertical semicircular canals may well turn out to be a titanosaurian synapomorphy. We have previously brought up the reasons behind the reduction of the semicircular canal radii of curvature in most sauropods [9,29,42], and quantitative analyses are underway. In birds, the slenderness and great length of the semicircular canals is related *inter alia* with their level of exposure to abrupt displacements during flight [53]. The reduction of the relative length of the semicircular canals in titanosaurians (an observation in line with the above-mentioned reduction of the floccular recess) and other sauropods might be linked to a restricted head rotation range.

Among the few titanosaurians whose inner ear has been described, the vestibular apparatus of MCCM-HUE-1667 resembles that of MCF-PVPH-765 ([8]: fig. 9C, G; [11]: fig. 3). The rostral semicircular canal is somewhat longer than the caudal. In contrast, the lateral semicircular canal is notably shorter. The angle between the rostral and lateral semicircular canals is about 110°, which is notably greater than the condition seen in *Ampelosaurus* sp. ([9]: figs S1, S2, S3). The two vertical semicircular canals diverge at somewhat less than 90°, which is as in *Bonatitan reigi* ([8]: fig. 9E), but greater than in *Ampelosaurus* sp. ([9]: figs S1, S2, S3) and MCF-PVPH-765 ([11]: fig. 3C) and a little less than in *Antarctosaurus wichmannianus* ([8]: fig. 9F). The angle between the caudal and lateral semicircular canals is about 90°, which is slightly less than what is seen in *Ampelosaurus* sp. ([9]: figs S1, S2, S3).

The lagena (= cochlea) is slightly arched medially (Fig 7B, S1 Fig), following the curvature of the internal surface of the braincase cavity at this place. It is irregularly ellipsoid in cross section; its ventral extremity is blunt (Fig 7A and 7B, S1 Fig). The length of the lagena is relatively greater than in many other titanosaurians, being much longer than in CCMGE 628/12457 ([10]: fig. 4A-C; Fig 8) and *Jainosaurus septentrionalis* ([10]: fig. 4D-F). The oval window is unevenly round in outline (Fig 7A, S1 Fig). It faces laterally and lies at the medial end of a relatively long duct, which reflects the thickness of the bony wall separating the labyrinth from the middle ear (Fig 7A and 7B, S1 Fig). At about the same height as the oval window, but more medially, the perilymphatic duct heads caudally (Fig 7B, S1 Fig), towards the metotic canal, which it reaches after a short course. From there on, it cannot be distinguished from the other structures that pass across the metotic foramen (see above). The perilymphatic duct is ellipsoid in outline as it enters the metotic group (Fig 7B, S1 Fig).

## Discussion

Skeletal remains of sauropods are generally common fossils in the continental Campanian and Maastrichtian of Europe, from Spain to Romania, but braincases, which are delicate bone complexes, are rare components of this record. Even though the number of species represented remains unclear, our knowledge of the diversity of European latest Cretaceous sauropods has improved recently. For instance, no less than eight “forms,” possibly representing as many species, have been distinguished in the Late Campanian-Late Maastrichtian interval of southwestern Europe based on femoral morphology [54]. This record, which is intriguing from a biogeographical and ecological perspective, includes in the Late Campanian alone *Lirainosaurus astibiae* [55], *Ampelosaurus atacis* [56], *Atsinganosaurus velauciencis* [57], and an indeterminate taxon. Even if neurocranial and postcranial elements are not found preserved together or in articulation for all these forms in further excavations, a pattern of co-occurrence should materialize in the long run, allowing the matching of dental, cranial, and postcranial elements.

Much progress has been accomplished recently regarding the phylogeny of basal titanosauriforms [58–60]. Unfortunately, this does not hold true for more derived taxa. This situation is exacerbated for both the European species, such as *Lirainosaurus astibiae* and *Ampelosaurus atacis*, as well as *Jainosaurus septentrionalis*, which are rarely taken into account in cladistic analyses. Thus, even the supertree depicting the phylogenetic relationships for titanosauriforms produced by Souza and Santucci [61], which is based on source trees selected to maximize the number of taxa in the analysis, does not include any of these three species as terminal taxa. As a result, there is no suitable recent phylogeny onto which the characters of MCCM-HUE-1667 could be optimized. Nonetheless, MCCM-HUE-1667 shows features that are informative for determining its relationships.

It has been proposed that the ratio of the mediolateral width of the basal tubera to that of the occipital condyle may present a useful phylogenetic character [62], a high ratio (1.5 or greater) possibly being synapomorphic of Lithostrotia [59]. Characters based on ratios with arbitrary cut-offs are problematic in some ways, and thus we do not want to place too much importance on it, but nevertheless, in MCCM-HUE-1667, this ratio is estimated to be about 1.88. This value is close to that of *Jainosaurus* (1.91; [62]: tab. 1), and may support lithostrotian affinities for MCCM-HUE-1667. Lithostrotia is the least inclusive clade containing *Malawisaurus dixeyi* and *Saltasaurus loricatus* ([63]: 311) and, therefore, represent a large group of derived titanosaurians. However, MCCM-HUE-1667 also shows a number of characters that are not widely distributed in the data matrix of Curry Rogers [64] and may suggest more refined, less-inclusive groupings. For example, MCCM-HUE-1667 has a supraoccipital that broadly contacts the caudal border of the parietal as in *Antarctosaurus* and *Jainosaurus*, which is not true for *Nemegtosaurus*, *Quaesitosaurus*, *Rapetosaurus*, and others. Likewise, MCCM-HUE-1667 has a flat occiput similar to that of *Ampelosaurus* and *Jainosaurus*, but it is not the case in *Malawisaurus*, *Nemegtosaurus*, *Quaesitosaurus*, *Rapetosaurus*, *Saltasaurus*, and others. Curry Rogers [64] scored *Antarctosaurus* as having the occipital region rostrocaudally deep with the paroccipital processes oriented caudolaterally. However, the paroccipital processes of *Antarctosaurus* ([21]: pl. 28 fig. 2; [22]: fig. 64 fig. e) extend much more laterally than caudally, conferring a fairly flat occiput to this taxon. In fact, in caudal view the basicranium of MCCM-HUE-1667, including the basal tubera and basipterygoid processes, resembles quite remarkably that of *Antarctosaurus* ([21]: pl. 28 fig. 1; [22]: fig. 64 fig. a) and other titanosaurian specimens from Argentina, such as the indeterminate braincase (MUCPv-334) of possible Santonian age described by Calvo and Kellner ([65]: fig. 1). Furthermore, the resemblances between MCCM-HUE-1667 and *Jainosaurus* ([28]: figs 3–7) are so marked, both in neurocranial osteology (e.g., shape and orientation of the paroccipital process) and endocast morphology (e.g., outline in lateral view), that a close phylogenetic proximity is suggested. *Jainosaurus* is now considered as pertaining to a clade of advanced titanosaurians together with other Gondwanan taxa such as *Antarctosaurus*, with which it shares a number of putatively derived characters such as a sinuous parietal-supraoccipital contact [28]. The latter character is also present in MCCM-HUE-1667. We surmise that MCCM-HUE-1667 would represent a derived titanosaurian probably allied to *Jainosaurus* and *Antarctosaurus*.

It is difficult to draw firm conclusions from the variety observed in the titanosaurian braincases found so far in the Upper Cretaceous of Europe, because of challenges associated with teasing apart interspecific from intraspecific variations, as well as the major problem of associating isolated braincases with other skeletal specimens. Caveats aside, analyzing and publishing these isolated specimens has the benefit of providing the basis for testing future hypotheses of association as more complete specimens are discovered. Undoubtedly, more than a few species dwelled that area at that time. Although the large number of titanosaurian elements from “Lo Hueco” (many of which are in articulation) are yet to be fully prepared and described, preliminary observations suggest that at least two species are present, which can be differentiated by the robustness of their skeleton and a variety of dental and postcranial characters (see in particular [66]). The examination of the neurocranial material ([9]; this work) supports this view as several features of the new specimen (e.g., ellipsoid foramen magnum) clearly show that the differences between the two titanosaurian braincases from “Lo Hueco” are not a product of the certain postmortem deformation suffered by MCCM-HUE-1667, but instead that the latter does not pertain to the same species as *Ampelosaurus* sp. The braincases alone reveal that *Ampelosaurus*-like and *Jainosaurus*-like forms were present in the “Lo Hueco” area around the Campanian-Maastrichtian boundary. The latter might represent a more derived form than *Ampelosaurus* sp. if we are to judge from the degree of offsetting of the course of the abducens nerve. Indeed, the more medial route of this nerve in *Ampelosaurus* sp. compared to MCCM-HUE-1667 is closer to the primordial condition in titanosauriforms and other sauropods in which this nerve passes through the pituitary fossa.

## Supporting Information

S1 Fig Interactive visualization made from the CT scan of the indeterminate titanosaurian sauropod braincase MCCM-HUE-1667 from the Cretaceous of Fuentes, Spain. (PDF)
