# Supplementary figures and images for: A New Titanosaurian Braincase from the Cretaceous “Lo Hueco” Locality in Spain Sheds Light on Neuroanatomical Evolution within Titanosauria
Source: PLoS One. 2015 Oct 7;10(10):e0138233. doi: 10.1371/journal.pone.0138233 (PMC4596832; doi:10.1371/journal.pone.0138233)

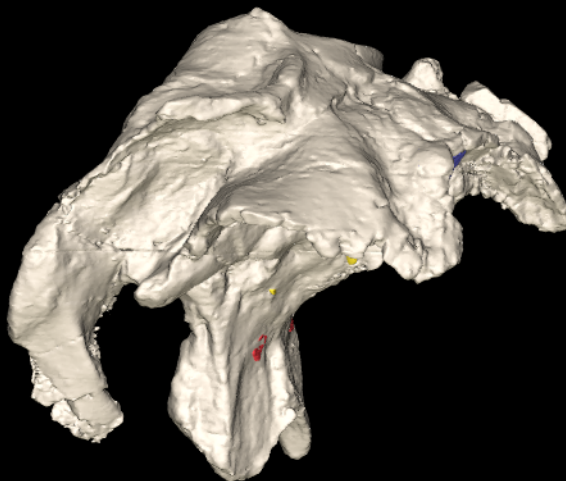

Supplement: S1 Fig — (PDF) [file pone.0138233.s001.pdf]
